# Manual versus semiautomatic segmentation of soft-tissue sarcomas on magnetic resonance imaging: evaluation of similarity and comparison of segmentation times

**DOI:** 10.1590/0100-3984.2020.0028

**Published:** 2021

**Authors:** Fernando Carrasco Ferreira Dionisio, Larissa Santos Oliveira, Mateus de Andrade Hernandes, Edgard Eduard Engel, Paulo Mazzoncini de Azevedo-Marques, Marcello Henrique Nogueira-Barbosa

**Affiliations:** 1 Hospital das Clínicas da Faculdade de Medicina de Ribeirão Preto da Universidade de São Paulo (HCFMRP-USP), Ribeirão Preto, SP, Brazil.; 2 Faculdade de Medicina de Ribeirão Preto da Universidade de São Paulo (FMRP-USP), Ribeirão Preto, SP, Brazil.

**Keywords:** Image processing, computer-assisted, Sarcoma/diagnostic imaging, Soft tissue neoplasms/diagnostic imaging, Magnetic resonance imaging, Reproducibility of results, Processamento de imagem assistida por computador, Sarcoma/diagnóstico por imagem, Neoplasias de tecidos moles/diagnóstico por imagem, Ressonância magnética, Reprodutibilidade dos testes

## Abstract

**Objective:**

To evaluate the degree of similarity between manual and semiautomatic segmentation of soft-tissue sarcomas on magnetic resonance imaging (MRI).

**Materials and Methods:**

This was a retrospective study of 15 MRI examinations of patients with histopathologically confirmed soft-tissue sarcomas acquired before therapeutic intervention. Manual and semiautomatic segmentations were performed by three radiologists, working independently, using the software 3D Slicer. The Dice similarity coefficient (DSC) and the Hausdorff distance were calculated in order to evaluate the similarity between manual and semiautomatic segmentation. To compare the two modalities in terms of the tumor volumes obtained, we also calculated descriptive statistics and intraclass correlation coefficients (ICCs).

**Results:**

In the comparison between manual and semiautomatic segmentation, the DSC values ranged from 0.871 to 0.973. The comparison of the volumes segmented by the two modalities resulted in ICCs between 0.9927 and 0.9990. The DSC values ranged from 0.849 to 0.979 for intraobserver variability and from 0.741 to 0.972 for interobserver variability. There was no significant difference between the semiautomatic and manual modalities in terms of the segmentation times (*p* > 0.05).

**Conclusion:**

There appears to be a high degree of similarity between manual and semiautomatic segmentation, with no significant difference between the two modalities in terms of the time required for segmentation.

## Abstract (pt)

**Objetivo:**

Verificar a similaridade entre as segmentações manual e semiautomática de sarcomas de tecidos moles na ressonância magnética (RM) e a similaridade interobservador e intraobservador entre as segmentações manuais.

**Materiais e Métodos:**

Estudo retrospectivo que incluiu 15 exames de RM de pacientes com diagnóstico de sarcoma de tecidos moles realizados antes de intervenções terapêuticas. As segmentações manual e semiautomática foram realizadas por três radiologistas utilizando o *software* 3D Slicer. O coeficiente de similaridade Dice (CSD) e a distância de Hausdorff foram utilizados para avaliar a similaridade das segmentações. Análise estatística descritiva e coeficiente de correlação intraclasse (CCI) foram realizados para comparar volumes tumorais.

**Resultados:**

A comparação dos métodos manual e semiautomático obteve valores de CSD entre 0,871 e 0,973. A comparação dos volumes segmentados pelos dois métodos de segmentação mostrou CCI entre 0,9927 e 0,9990. As análises intraobservador e interobservador obtiveram valores de CSD, respectivamente, de 0,849 a 0,979 e de 0,741 a 0,972. Não houve diferença significativa entre os tempos de segmentação dos métodos semiautomático e manual (*p* > 0,05).

**Conclusão:**

Houve alta similaridade entre as segmentações de sarcomas de tecidos moles obtidas pelos métodos manual e semiautomático, sem diferença significativa para o tempo despendido para as segmentações.

## INTRODUCTION

Soft-tissue sarcomas are a heterogeneous group of malignant tumors with a broad spectrum of histological presentations and prognoses^(1)^. Although they affect connective tissues throughout the body, the most common location is in the extremities, in 59% of cases, followed by the trunk, in 19%, the retroperitoneum, in 15%, and the head/neck region, in 9%^(2,3)^.

Soft-tissue sarcomas are responsible for approximately 7-15% of malignant tumors in pediatric patients and approximately 1% of those in adults^(2)^. Magnetic resonance imaging (MRI) is the recommended method for assessing soft-tissue tumors, because it has excellent contrast resolution, does not use ionizing radiation, and allows multiplanar image acquisition^(4)^.

Radiomics was proposed as a way to extract a large amount of quantitative information from the imaging examinations available in the clinical routine, as well as to integrate predictive models of diagnosis, prognosis, and therapeutic response^(5)^. Medical images contain measurable information that may reflect the pathophysiology of the disease and that can be revealed via quantitative analysis^(6)^. The segmentation process, which consists of marking the region of interest from which the quantitative information will be extracted for analysis, is essential to the application of radiomics. There are three types of segmentation: manual, semiautomatic, and automatic. Manual segmentation consists in segmenting the lesion or anatomical region of interest by using a graphical interface, such as a mouse or digital pen, with which the user marks the limits of the lesion or the segmented organ, a time-consuming process that requires knowledge of the sectional anatomy in the images. Semiautomatic segmentation consists in using software with segmentation algorithms that delineate the lesion on the basis of information initially provided by the user in a manual process. In general, it may be necessary to make manual adjustments after the selection has been made. Automatic segmentation consists in completely automated identification of the contours of the lesion by the software, without the use of data provided through a manual process. Although automatic segmentation appears to be more effective, especially considering its time-saving potential, it requires complex computational resources and, in general, it is not easy to obtain satisfactory results^(7)^.

In a search of the literature, we identified various studies that analyzed MRI scans of soft-tissue sarcomas using manual segmentation^(8-15)^, semiautomatic segmentation^(16,17)^, or automatic segmentation^(18)^. Only one of those studies evaluated interobserver variability^(13)^, and none of them evaluated intraobserver variability. More importantly, we identified no studies evaluating the similarity between manual and semiautomatic segmentation methods in soft-tissue sarcomas, a gap in the current literature that motivated us to carry out the present study. We were also motivated by the need to evaluate manual and semiautomatic segmentation methods in general and the possibility of reducing segmentation time.

The objective of this study was to evaluate the similarity between manual and semiautomatic segmentation of soft-tissue sarcomas on MRI scans. An additional objective was to evaluate intraobserver and interobserver variability in manual segmentations.

## MATERIALS AND METHODS

The study was approved by the research ethics committee of the university hospital where it was conducted. Because the study involved the retrospective analysis of images, the requirement for written informed consent was waived. All patient information contained in the Digital Imaging and Communication in Medicine (DICOM) files related to the MRI scans was anonymized using the K-PACS viewer (IMAGE Information Systems, Rostock, Germany). To ensure patient privacy, the files were identified by numbers (patient 1, patient 2, etc.) and not by name or initials.

### Selection of cases and images

All of the examinations evaluated were performed in a 1.5-T scanner (Achieva; Philips Medical Systems, Eindhoven, The Netherlands). We selected cases of examinations performed between January 2006 and January 2016 that were in the radiology database of our institution, searching for the keyword “sarcoma” in the conclusion section of MRI reports.

For segmentation, we selected images acquired in the axial plane in T2-weighted sequences with fat suppression. The mean acquisition parameters were as follows: repetition time (TR) of 4316 ms (range, 3489- to 4733 ms); echo time (TE) of 55 ms (range, 50-60 ms); matrix of 563 × 563 pixels (range, 320 × 320-864 × 864 pixels); slice thickness of 5.2 mm (range, 3-7 mm); and spatial resolution of 0.571 mm/pixel (range, 0.234-0.742 mm/pixel). In one case (a well-differentiated liposarcoma), we selected a T1-weighted sequence without fat suppression, with a TR of 352 ms, a TE of 10 ms, matrix of 480 × 480 pixels, a slice thickness of 6.0 mm, and a spatial resolution of 0.686 mm/pixel.

**Figure 1:**
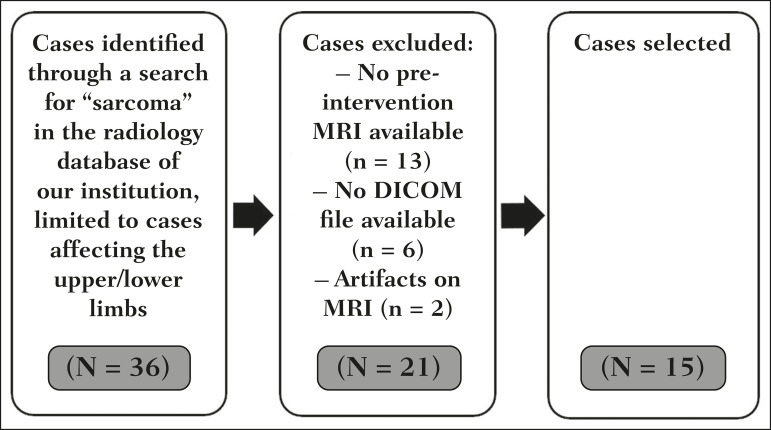
Case selection flow chart.

For the selection of cases to be included in the study, we applied the following inclusion criteria: the diagnostic confirmation of soft-tissue sarcoma by histopathological study being included in the hospital database; the tumor being located in the appendicular skeleton (upper or lower limbs); the MRI examination files being available in DICOM format; and the MRI examination having been performed at our institution, prior to any diagnostic or therapeutic intervention (biopsy, surgery, chemotherapy, or radiotherapy). Cases in which movement artifacts impeded the analysis of the images were excluded. Figure 1 summarizes the selection process.

### MRI analysis and segmentation

Manual and semiautomatic segmentation were performed using freeware for processing medical images (3D Slicer, version 4.6.2; https://www.slicer.org). Semiautomatic segmentation of MRI scans was performed with the 3D Slicer tool GrowCut. Segmentations were performed on images acquired in the axial plane via T2-weighted imaging (T2WI) with fat suppression. In one case (of liposarcoma), the segmentation process involved images acquired in the axial plane via T1-weighted imaging (T1WI) without fat suppression.

**Figure 2:**
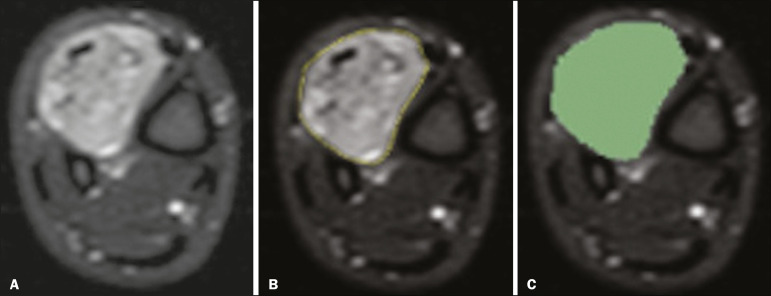
Steps of manual segmentation. **A**: Tumor identification. **B**: Delineation of tumor boundaries (yellow line). **C**: Creation of the segmented volume of interest in a slice of the tumor (green area).

Three radiologists, working independently and blinded to the other segmentations and histopathological results, performed manual segmentation of the sarcomas. Two of those radiologists (designated radiologist 1 and radiologist 2, respectively) were fellows in musculoskeletal radiology, and the third (designated radiologist 3) had five years of experience in the field. Manual segmentation was performed by manually delineating the tumor borders in each sectional slice, in all sectional slices in which tumor tissue was present (Figure 2), according to procedures followed in previous studies^(13,19-23)^. Radiologist 1 manually segmented all cases again after a one-month interval, which allowed us to assess intraobserver variability. Interobserver variability was evaluated by comparing the segmentations performed by the three radiologists.

**Figure 3:**
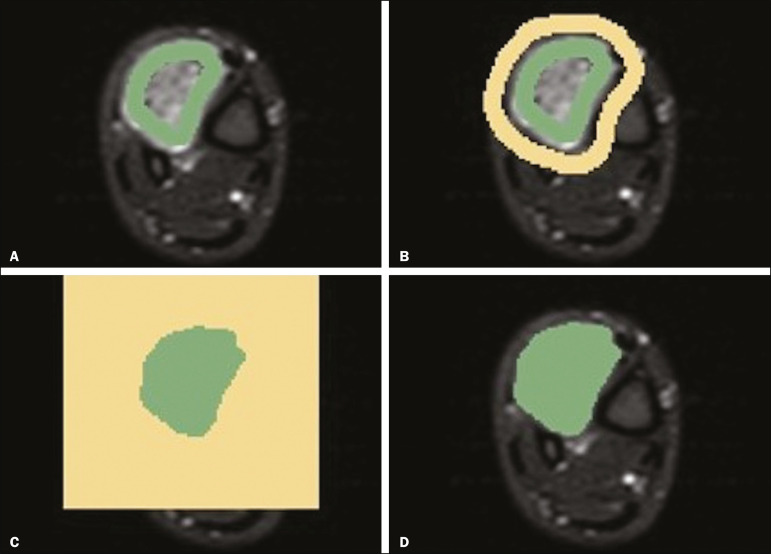
Steps of semiautomatic segmentation. **A**: Marking part of the internal region of the tumor (in green). **B**: Marking part of the external region adjacent to the tumor (in yellow). **C**: Segmentation of the internal and external areas of the tumor by the GrowCut tool (in green and yellow, respectively). **D**: Selection of the segmented internal area of the tumor to create the volume of interest.

For the semiautomatic segmentation process, performed with the 3D Slicer GrowCut tool (Figure 3), the user roughly identifies regions within the tumor tissue and adjacent (non-neoplastic) tissues. Thereafter, the software delineates a volume of interest within the segmented lesion. It is possible to make manual corrections to the contours obtained by the software. There are various algorithms for semiautomatic segmentation, and our method is consistent with that proposed by Egger et al.^(19,20)^, who also employed the GrowCut tool. Radiologist 1 performed semiautomatic segmentation two months after the second manual segmentation, and another analysis was performed to compare the manual and semiautomatic segmentations.

In the present study, for both segmentation methods (manual and semiautomatic), the segmented volume of interest represented the total extent of the tumor. The volume did not include the area of peritumoral edema. Another segmentation criterion was to include neurovascular or tendon structures in the segmented area when those anatomical structures showed circumferential (360°) involvement by the neoplasm in the axial plane.

In our study, we chose fat-suppressed axial T2WI as the standard for performing segmentations, because that sequence was available in all cases. In cases in which the axial T2WI sequence with fat suppression showed low tissue contrast between the tumor and the surrounding tissues, the T1WI sequence was available and was used to assist in the segmentation process.

The time required for the manual and semiautomatic segmentations performed by radiologist 1 was recorded for later comparison, with a digital timer controlled by another researcher who was not performing the segmentation. In the manual segmentation time counting, the images were opened in the 3D Slicer software to obtain a quick overview of the tumor. The timer was started immediately before the start of the process of delineating the tumor contours and was stopped after the delineation of the tumor contours in the last sectional slice that contained tumor tissue. In the semiautomatic segmentation, the images were opened in the 3D Slicer software and a quick overview of the tumor was obtained, the central sectional slice of the tumor being identified after the total number of slices containing tumor tissue had been counted. The timer was started immediately before the manual delineation of the tumor and of the tissue external to the tumor in the central sectional slice and was stopped after manual corrections had been made to the segmentation map generated by the GrowCut algorithm. That standardization followed the example of previous segmentation studies employing the 3D Slicer software and the GrowCut tool in the analysis other histological types of tumors^(19,20)^.

### Statistical analysis

The Dice similarity coefficient (DSC) and the Hausdorff distance (HD) were used in order to assess the similarity between manual and semiautomatic segmentation, as well as to evaluate the intraobserver and interobserver variability in manual segmentations. The 3D Slicer software obtains the DSC and HD by comparing the segmentations performed.

The DSC measures the spatial overlap, ranging from 0 to 1, and can serve as a metric for validating the similarity between two segments. A value of 0 indicates that there is no spatial overlap, whereas a value of 1 indicates that there is total overlap of the segments. We categorized the DSC result as follows: < 0.7 = low similarity; 0.7 ≤ < 0.8 = good similarity^(24-27)^; and ≥ 0.8 = high similarity^(28)^.

The HD between two segmented volumes is defined in terms of the Euclidean distance between their boundary voxels. For example, when the semiautomatic segmentation edge voxels and the manual segmentation edge voxels are considered, the maximum HD is defined as the maximum Euclidean distance between any of the points in the first group and the points of the second group^(20)^.

Comparisons were also made of the tumor volumes obtained in the segmentations. Means, standard deviations, and intraclass correlation coefficients (ICCs) were obtained from the segmented volumes. Statistical comparisons of volumes were performed in different situations to determine whether there were statistically significant differences among them.

The times for manual and semiautomatic segmentation performed by radiologist 1 were compared; the means and standard deviations were obtained; and the Wilcoxon test was applied to identify statistical differences in the segmentation execution time between the two methods. In addition, we used Spearman’s correlation coefficient to quantify the correlation between the segmentation time and the tumor volume to be segmented.

## RESULTS

After searching the radiology database of our institution for the term “sarcoma”, we identified 36 examinations in which a sarcoma was located in an extremity. We analyzed the examinations and searched for clinical information in the hospital database, thus finding that there were 13 cases in which the MRI examination was not performed prior to an invasive therapeutic or diagnostic intervention. Of the remaining 23 examinations, six were not available in DICOM format and two presented artifacts, those eight examinations therefore being excluded. Thus, we selected 15 examinations acquired in 15 different patients, of whom eight were female and seven were male. The mean age was 50.8 years (range, 6-91 years). Although all of the patients had a confirmed diagnosis of soft-tissue sarcoma, there were seven different histological subtypes, the most common being liposarcoma, which was identified in five patients. The location of the tumors in the limbs varied, the thigh being the site most often affected (in five cases).

Table 1 shows the epidemiological data related to the cases evaluated in this study, including the histopathological subtypes of the tumors.

**Table 1:** Distribution of cases of soft-tissue sarcoma by gender, age, location, and histopathological type of the tumor.

Patient	Gender	Age (years)	Location	Histopathological type
1	Female	6	Right leg	Extraosseous Ewing's sarcoma
2	Female	13	Right leg	Synovial sarcoma
3	Male	61	Pelvis and upper left thigh	Grade II myxoid sarcoma
4	Male	45	Right elbow	Grade II myxoid sarcoma
5	Male	30	Upper right hemithorax	High-grade sarcoma
6	Male	77	Right thigh	Well-differentiated liposarcoma
7	Female	57	Right forearm	Myxoinflammatory fibroblastic sarcoma
8	Male	64	Left knee	High-grade pleomorphic sarcoma
9	Female	87	Left thigh	High-grade undifferentiated myxoid pleomorphic sarcoma
10	Male	40	Left thigh	Well-differentiated liposarcoma
11	Male	58	Left thigh	High-grade myxoid liposarcoma
12	Female	73	Right leg	High-grade undifferentiated pleomorphic sarcoma
13	Female	19	Left leg	Myxoid liposarcoma
14	Female	91	Right hand	Poorly differentiated fibrosarcoma
15	Female	42	Right gluteal region	Myxoid liposarcoma

### Manual segmentation

#### Intraobserver variability

In the comparison of the two manual segmentations performed by radiologist 1, the DSC ranged from 0.849 to 0.979 and the HD ranged from 3.53 mm to 20.96 mm. The comparison between the volumes of the two manual segmentations resulted in an ICC of 0.999. Table 2 summarizes the analysis of intraobserver variability for the two manual segmentations.

**Table 2:** Descriptive statistics for the DSC and HD data obtained in the comparison between the first and second manual segmentations performed by radiologist 1.

Statistic	DSC	HD (mm)
Minimum	0.849	3.53
Maximum	0.979	20.96
Mean ± SD	0.947 ± 0.03	10.3 ± 5.66
Coefficient of variation	3.8	56.4
Confidence interval	0.927-0.968	6.89-13.16

The time required for manual segmentation of the cases ranged from 2 to 32 min, with a mean of 12.4 ± 7.73 min. The Spearman’s correlation coefficient obtained for the comparison between the tumor volume and the manual segmentation time was 0.59 (*p* = 0.020), indicating that the association between the two was statistically significant.

#### Interobserver variability

The manual segmentations performed by the three radiologists were compared with each other. For the comparison between the manual segmentations performed by radiologists 1 and 2, the DSC ranged from 0.781 to 0.973 and the HD ranged from 5.83 mm to 27.81 mm. The comparison between the manual segmentations performed by radiologists 1 and 3 resulted in DSC values ranging from 0.741 to 0.972 and HD values ranging from 8.08 mm to 61.84 mm. Tables 3 and 4 summarize the analysis of the interobserver variability for the manual segmentations.

**Table 3:** Descriptive statistics for the DSC and HD data obtained in the comparison of the manual segmentation performed by radiologist 1 and that performed by radiologist 2.

Statistic	DSC	HD (mm)
Minimum	0.781	5.83
Maximum	0.973	27.81
Mean ± SD	0.917 ± 0.05	12.94 ± 7.12
Coefficient of variation	6.3	55.0
Confidence interval	0.884-0.949	9.00-16.89

**Table 4:** Descriptive statistics for the DSC and HD data obtained in the com- parison of the manual segmentation performed by radiologist 1 and that performed by radiologist 3.

Statistic	DSC	HD (mm)
Minimum	0.741	8.08
Maximum	0.972	61.84
Mean ± SD	0.891 ± 0.08	12.34 ± 12.83
Coefficient of variation	9.1	63.3
Confidence interval	0.845-0.936	12.23-26.44

The comparison between the volumes obtained in the manual segmentations performed by radiologists 1 and 2 resulted in an ICC of 0.9976. Similar results were obtained for the comparison between the volumes obtained in the manual segmentations performed by radiologists 1 and 3, with an ICC of 0.9927.

### Semiautomatic segmentation

The comparison between the manual and semiautomatic segmentations performed by radiologist 1 resulted in DSC values ranging from 0.871 to 0.973 and HD values ranging from 5.43 mm to 31.75 mm. The comparison between the volumes obtained in manual and semiautomatic segmentation resulted in an ICC of 0.9990. Table 5 summarizes the comparison between manual and semiautomatic segmentation. The time required for semiautomatic segmentation of the cases ranged from 7 to 34 min, with a mean of 13.8 ± 7.23 min. The Spearman’s correlation coefficient obtained for the comparison between the tumor volume and the semiautomatic segmentation time was 0.32 (*p* = 0.25), indicating that the association between the two was not statistically significant.

**Table 5:** Descriptive statistics for the DSC and HD data obtained in the com- parison of the manual and semiautomatic segmentations performed by radi- ologist 1.

Statistic	DSC	HD (mm)
Minimum	0.871	5.43
Maximum	0.973	31.75
Mean ± SD	0.947 ± 0.02	11.55 ± 7.61
Coefficient of variation	2.7	65.8
Confidence interval	0.933-0.962	7.34-15.77

### Comparison of segmentation time between manual and semiautomatic methods

In 9 of the 15 segmented cases, the manual segmentation time was shorter than was the semiautomatic segmentation time, although the difference was not statistically significant when evaluated by the Wilcoxon test (*p* > 0.05). The volumes and segmentation times for the manual and semiautomatic segmentations performed by radiologist 1 are shown in Table 6.

**Table 6:** Estimated volumes and measured segmentation times for the manual and semiautomatic segmentations performed by radiologist 1.

Order*	Tumor volume estimated by manual segmentation (cm^3^)	Tumor volume estimated by semiautomatic segmentation (cm^3^)	Manual segmentation time (min)	Semiautomatic segmentation time (min)
1	18.9	18.3	6	9
2	38.6	35.9	12	17
3	50.9	47.4	10	7
4	59.1	60.9	2	23
5	71.5	69.2	8	14
6	161.3	154.1	7	8
7	257.5	242.5	11	8
8	362.0	345.3	9	13
9	388.8	369.1	20	8
10	659.0	617.9	8	13
11	749.5	720.4	16	9
12	927,8	890.2	12	11
13	1136.7	1089.0	24	14
14	1218.7	1326.8	9	19
15	3486.9	3381.1	32	34

* Distribution of cases in ascending order by tumor volume.

## DISCUSSION

In the present study, we found high similarity between semiautomatic and manual segmentation of soft-tissue sarcomas on MRI examinations. However, the time savings expected to be associated with the semiautomatic method were not observed. For the manual segmentations, the level of intraobserver agreement was excellent (DSC ≥ 0.8) and the level of interobserver agreement was good (0.7 ≤ DSC <0.8). The comparison between the volumes segmented showed high similarity, underscoring the good reproducibility between the two methods.

Our results are consistent with those in the literature, which indicate some degree of similarity between manual and semiautomatic MRI segmentation of other neoplasms, such as glioblastoma multiforme^(19)^, pituitary adenoma^(20)^, and hepatocellular carcinoma^(27)^. Our findings are also in keeping with those of studies indicating good interobserver agreement in the manual segmentation of soft-tissue sarcomas on MRI scans, such as the study conducted by Peeken et al.^(13)^, in which an even better coefficient of similarity was obtained, with a mean DSC of 0.91 ± 0.069. However, those authors did not compare manual and semiautomatic segmentation, did not evaluate the reproducibility of the segmentation volumes, and included examinations of abdominal and retroperitoneal sarcomas.

The difference in tissue contrast among the various MRI sequences can affect the segmentation of the tumor, therefore having the potential to reduce the reproducibility of the method. In the case of soft-tissue sarcomas, this choice of sequences is not an easy task, because of the variety of histological subtypes and their potentially heterogeneous imaging characteristics. The literature indicates some variability in the types of sequences chosen for segmentation and radiomic analysis of such tumors^(8-16)^. Several recent studies that applied radiomics to soft-tissue sarcomas used segmentation in T2WI sequences in a manner similar to that of our study, some of them also segmenting other sequences for specific analyses^(8,12-15)^. In some studies of radiomic analysis of soft-tissue sarcomas on MRI, the segmentation was obtained in a specific sequence, although other sequences were available for analysis^(9,11,12)^. We chose to use axial T2WI sequences because they were available in all cases and because they provide high tissue contrast between a neoplasm and the adjacent soft tissues. In one case (of a well-differentiated liposarcoma), we also used a T1WI sequence without fat suppression, which helped us delineate the tumor because it allows such tumors to be more easily visualized. Although there were five cases of liposarcoma in our study sample, only that one case involved a well-differentiated liposarcoma with margins that were sufficiently distinct to be delineated in T2WI sequences with fat suppression. The others had a predominance of myxoid tissue or were of high grade and had contours that were sufficiently delineated for segmentation to be performed in the T2WI sequences. Hypothetically, a T1WI sequence without fat suppression could provide greater benefit in the segmentation of liposarcomas, especially those that are more well differentiated histologically. However, the design of our study did not allow us to compare the specific influence that the use of different sequences has on the segmentation similarity results. One study that specifically analyzed the differentiation between lipomas and liposarcomas employed segmentation of T1WI sequences, underscoring the importance of their use in the evaluation of lipomatous tumors, although the authors did not evaluate the reproducibility of the segmentation itself^(16)^.

Vallières et al.^(8)^ performed manual segmentation to delineate the contours of soft-tissue sarcomas. In cases in which perilesional edema was visible, the authors performed an additional segmentation incorporating the area of the edema, although they did not evaluate the reproducibility of the additional segmentation. As previously mentioned, we excluded peritumoral edema from the segmentation in our sample of cases. The potential importance of the area of edema is related to the possible presence of tumor cells^(29)^. In clinical practice, however, perilesional edema is not always surgically resected^(29)^. Multiple studies involving radiomic analysis of soft-tissue sarcomas also did not include peritumoral edema^(9-13,15)^, which underscores the potential applicability of our results. To date, there have been no specific studies evaluating the importance of peritumoral edema in the radiomic analysis of cases of bone sarcoma, nor have there been any evaluating the reproducibility of the segmentation of such edema. Because edema usually has a poorly defined aspect, it can presumably pose a challenge for obtaining a high degree of similarity in segmentation. In cases of brain tumors and cranial meningiomas^(30,31)^, a high degree of similarity in the segmentation of peritumoral edema was demonstrated with semiautomatic methods. However, to our knowledge, there have been no studies specifically evaluating the similarity of the segmentation of peritumoral edema in cases of soft-tissue sarcoma. There is a need for further studies to bridge the gap in the literature regarding the segmentation of peritumoral edema in soft-tissue sarcomas.

The time required for manual segmentation can be influenced by several factors, the main ones being the volume of the tumor and the delineation of its margins^(32,33)^. The larger the tumor is, the greater will be the number of sectional slices analyzed and segmented, which tends to increase the time required for segmentation. Our results show that there was a statistically significant correlation between the volume of the tumor and the time spent on manual segmentation, which was longer for tumors with greater volume. The difficulty in delineating the tumor is greater if its margins are poorly defined and if there is low tissue contrast between the tumor and the adjacent tissues, which makes the segmentation of such neoplasms challenging, especially that of those that present a heterogeneous aspect on imaging examinations^(6,7)^. In our sample, there were cases in which the sarcoma had poorly defined margins, and the semiautomatic segmentation therefore extrapolated the segmentation limits, including tissues that did not belong to the tumor. In those cases, segmentation time were longer because it was necessary to correct the contours of the tumor and the time spent for correction canceled out the potential time saving that is usually associated with the use of semiautomatic segmentation.

Despite the potential variability between manual segmentations performed by specialists, they are considered the gold standard for the comparison between automatic and semiautomatic methods^(34)^. In cases where there is little contrast between a neoplasm and the adjacent tissues, semiautomatic segmentation is also more likely to obtain results that do not represent the correct delineation of a sarcoma, which requires many manual adjustments by the user, increasing the total segmentation time. That occurred in one case in our sample, in which there was an infiltrative fibrosarcoma, with poorly defined margins, in the region of the hand.

The correlation obtained between the tumor volume and the time spent on manual segmentation confirms that tumor volume is a factor that influences that segmentation method, more time being required in order to segment larger tumors. In the case of semiautomatic segmentation, the segmentation time did not correlate significantly with the tumor volume. Therefore, other factors must have a significant impact on the segmentation time when the semiautomatic method is adopted. As previously mentioned, we believe that in the case of semiautomatic segmentation, tumors with poorly defined contours require more time for correction by the radiologist. It is recognized that algorithms based on continuous growth of manually demarcated regions tend to result in oversegmentation; that is, segmentations that include areas beyond the true limits of the structure to be segmented, especially for lesions or structures with ill-defined borders^(7)^. To our knowledge, there have been no studies comparing tumors with ill-defined contours and those with well-defined contours, in terms of the time required for their segmentation. We suggest that this is an interesting possibility for future research.

To our knowledge, there have been no studies standardizing the measurement of segmentation times in cases of soft-tissue sarcoma. Our finding that there was no statistically significant difference between the semiautomatic and manual segmentation methods, in terms of the mean segmentation time, differs from those of studies of other types of neoplasms. Egger et al.^(19,20)^ demonstrated that semiautomatic segmentation would be faster than manual segmentation in glioblastomas and pituitary adenomas, which are tumors of the central nervous system. Dioní sio et al.^(23)^ described the time-saving advantage of the semiautomatic method over the manual method for the segmentation of bone sarcomas on MRI. We assume that our results did not reproduce those of studies of brain tumors and bone sarcomas, given the considerably greater tissue heterogeneity of the soft-tissue sarcomas included in our study sample; in the present study, semiautomatic segmentation methods tended to necessitate greater manual correction of the results of semiautomatic segmentation obtained by the software, as discussed in a previous study^(32)^. The GrowCut segmentation algorithm is based on the identification of similarities in shades of gray between pixels at the edge of the lesion and pixels that are external but adjacent to the edge. When a tumor is heterogeneous, there is a greater probability that the periphery of the neoplasm will present pixels with signal intensity similar to that of tissues external to the tumor, extending the semiautomatic segmentation to external regions that do not belong to the neoplastic lesion. Thus, for some soft-tissue tumors, more time may be necessary, because of the need for manual correction to arrive at the correct final segmentation of the tumor. There is no universally accepted, satisfactory segmentation algorithm for all types of medical images^(7)^.

In our sample, we obtained manual segmentation times that were shorter than the semiautomatic segmentation times in 9 of the 15 cases. Although we detected no statistically significant difference between the manual and semiautomatic methods, in terms of the segmentation time, we hypothesize that the use of the semiautomatic method could reduce segmentation times in cases of larger sarcomas with well-defined contours. Our small sample size prevented us from performing a robust statistical evaluation to test that hypothesis. Therefore, there is a need for further studies to investigate that possibility.

Our study has some limitations. First, it was a retrospective study and included a relatively small number of cases. Our institution is a referral center for the treatment of sarcomas and other soft-tissue tumors; however, some patients who are referred from other facilities have previously undergone MRI studies elsewhere, and such cases were not included in our sample. Another limitation is the fact that only one radiologist performed semiautomatic segmentation and that we therefore had only one semiautomatic segmentation time data set to compare with the manual segmentation time data.

The DSC is a statistical tool designed to assess the degree of similarity between two samples or two sets of data, making it possible to identify the overlap between the two segmentations, and is currently the most widely used tool for measuring the similarity between different segmentations performed on medical images. The DSC can therefore be used in order to infer the similarity between the segmentations performed by different examiners or to calculate the similarity between the segmentation performed with a technique considered the gold standard and that performed with another technique.

There is no consensus in the literature on specific standardization for the interpretation of similarity results obtained by the DSC in segmentation studies^(35)^. Some authors have used a DSC value of 0.70 as a lower limit to consider good similarity between segmentations^(24-27)^. Zijdenbos et al.^(26)^ and Fleiss et al.^(36)^ considered the DSC as a special type of kappa statistic, stating that DSC values above 0.7 would be equivalent to a kappa above 0.75 and could be considered indicative of excellent similarity^(37)^. In contrast, Fontina et al.^(35)^ warned that the kappa statistic limits defined by Landis et al.^(38)^ were originally introduced for categorical data and that the use of the same parameters of interpretation of the kappa statistic for the analysis of the DSC could overestimate the similarity between segmentations. Mattiucci et al.^(28)^ suggested a DSC value of 0.8 as a lower limit to indicate high similarity between segmentations performed by different specialists.

Our results in assessing the similarity between manual and semiautomatic segmentation of soft-tissue sarcomas may be important in the context of MRI-based radiomics. For the manual segmentation of soft-tissue sarcomas in our sample, the level of intraobserver variability was excellent (DSC ≥ 0.8) and that of interobserver variability was good (0.7 ≤ DSC < 0.8). We suggest that further studies be conducted to evaluate semiautomatic segmentation tools designed to reduce time and effort in the segmentation process, since given that we were unable to obtain a reduction in segmentation time with the semiautomatic segmentation tool used.

## CONCLUSION

The results obtained in the present study indicate that there is a high degree of similarity between manual and semiautomatic segmentations, as well as that the use of the semiautomatic method does not appear to reduce the segmentation time for soft-tissue sarcomas. We also found that, when the 3D Slicer software is employed, there is, for manual segmentations, excellent intraobserver variability and good interobserver variability.
